# A Verified Implementation of Algebraic Numbers in Isabelle/HOL

**DOI:** 10.1007/s10817-018-09504-w

**Published:** 2018-12-09

**Authors:** Sebastiaan J. C. Joosten, René Thiemann, Akihisa Yamada

**Affiliations:** University of Innsbruck, Innsbruck, Austria

**Keywords:** Theorem proving, Algebraic numbers, Real algebraic geometry, Resultants

## Abstract

We formalize algebraic numbers in Isabelle/HOL. Our development serves as a verified implementation of algebraic operations on real and complex numbers. We moreover provide algorithms that can identify all the real or complex roots of rational polynomials, and two implementations to display algebraic numbers, an approximative version and an injective precise one. We obtain verified Haskell code for these operations via Isabelle’s code generator. The development combines various existing formalizations such as matrices, Sturm’s theorem, and polynomial factorization, and it includes new formalizations about bivariate polynomials, unique factorization domains, resultants and subresultants.

## Introduction

*Algebraic numbers*, i.e., the numbers that are expressed as roots of non-zero integer (or equivalently rational) polynomials, are an attractive subset of the real or complex numbers. Every satisfiable polynomial constraint has solutions in the domain of algebraic numbers; in particular, algebraic numbers are closed under arithmetic operations (addition, multiplication, integer powers, and there inverses). Moreover these arithmetic operations are precisely computable, and comparisons of algebraic numbers are decidable. As a consequence, algebraic numbers are an important utility in computer algebra systems; e.g., Collin’s cylindrical algebraic decomposition algorithm for solving problems in real algebraic geometry heavily relies upon algebraic numbers [20, Sect. 8.6.5].

Our original interest in algebraic numbers stems from a certification problem about automatically generated complexity proofs, where for a given matrix $A \in {\mathbb {Q}}^{n \times n}$ we have to compute the growth rate of $A^n$ for increasing *n* [25]. To this end, all complex roots of the characteristic polynomial of *A* have to be identified.

### Example 1

Consider a matrix *A* whose characteristic polynomial is $f(x) = \frac{1}{3} \cdot (1 + 2x + 3x^4)$ and let $\lambda _1,\dots ,\lambda _4$ be the complex roots of *f*. If the norm of some $\lambda _i$ is larger than 1, then the growth rate of *A* is exponential; if all norms are below 1, then $A^n$ tends to 0; and otherwise the growth rate is polynomial and its degree can be determined by further computations.

**Fig. 1:**
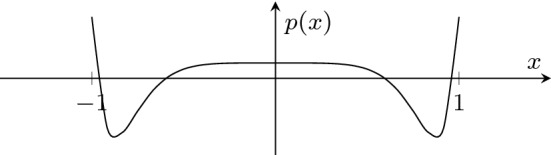
The polynomial *p* with its four real roots

In order to apply this criterion, we need to compute each norm $|\lambda _i| = \sqrt{ Re (\lambda _i)^2 + Im (\lambda _i)^2}$, and afterwards compare it with 1. All these computations can be performed with the help of algebraic numbers, as we will see throughout this paper. For instance, in this example we obtain the following results where roots are indexed from the smallest to the largest, and where the polynomials , , and  are constructed during the computation. 
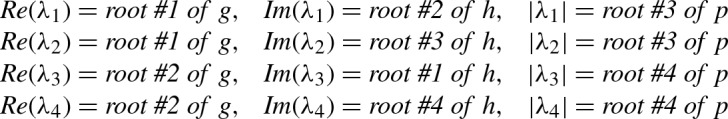
 As each norm $|\lambda _i|$ is below 1, cf. Fig. 1, we can conclude that $A^n$ tends to 0 for increasing *n*.

In this paper, we describe an implementation of algebraic numbers in Isabelle/HOL [21]. Up to our knowledge it is the first implementation that is both fully verified and executable on its own, i.e., without information from external tools. The implementation already became a crucial component of the automated reasoning tool CeTA for verifying complexity proofs [1, 25] that are generated during the annual termination competitions [12]. It is also used within a verified solver for linear recurrences [10].

The paper is structured as follows.

- We first introduce some basic notions of algebraic numbers and then formalize the fact that every algebraic number has a unique canonical polynomial that represents it. We argue that these canonical polynomials make a good internal representation (Sect. 2).
- For each algebraic operation, we formalize how to synthesize a polynomial that represents the output using polynomials that represent the inputs. We thus show that algebraic numbers are closed under the algebraic operations (Sect. 3).
- For the multiplication and addition of algebraic numbers, we refer to *resultants*. We implement and formalize the *subresultant remainder sequence algorithm*, which can efficiently compute resultants (Sect. 4).
- Using the above results, we implement the algebraic operations and comparisons of real and complex algebraic numbers, and a function to uniquely convert them into strings. We develop a hierarchy of four layers to represent real algebraic numbers, formalize several bisection algorithms, and integrate optimizations to obtain efficient code (Sect. 5).
- We moreover provide algorithms that identify all real and complex roots of a rational polynomial. Together with the fact that complex roots of a real polynomial come in complex conjugate pairs, we derive algorithms that completely factor rational polynomials into real or complex polynomial factors (Sect. 6).
- As we made an effort for efficiency, we experimentally compare the implementation against a version described in a preliminary version [24] of this paper, and the commercial computer algebra tool Wolfram Mathematica 11 (Sect. 7).

Most of the algorithms and proofs of our formalization are based on a textbook by Mishra [20, Chapters 7 and 8]; it contains a detailed implementation of real algebraic numbers, including proofs. When it comes to subresultants, we followed the original papers by Brown and Traub [2, 3]. However, our formalization also includes algorithms and optimizations, which we did not find in the literature, though they might be known.

For the Coq proof assistant, the Mathematical Components library^1^ contains various formalized results around algebraic numbers, e.g., quantifier elimination procedures for real closed fields [6]. In particular, the formalization of algebraic numbers for Coq is given by Cohen [4]. He employed Bézout’s theorem to derive desired properties of resultants, while we followed proofs by Mishra [20] and formalized various facts on resultants. Our work is orthogonal to the more recent work which avoids resultants [5]. A partial Coq formalization of subresultants also exists [19]. In contrast, our formalization is complete, and also integrates an optimization due to Ducos [8, Sect. 2].

For Isabelle, Li and Paulson [18] independently implemented algebraic numbers. They however did not formalize resultants; instead, they employed an external tool as an oracle to provide polynomials that represent desired algebraic numbers, and provided a method to validate that the polynomials from the oracle are suitable.^2^ Due to our optimization efforts, we can execute their examples [18, Fig. 3] in 0.016 seconds on our machine, where they reported 4.16 seconds.^3^

The whole formalization is available in the archive of formal proofs (AFP), mostly in entries Algebraic Numbers and Subresultants. Additionally, on

https://doi.org/10.5281/zenodo.1411394

we link statements in the paper with the Isabelle sources and provide details on our experiments.

## Representation of Algebraic Numbers

Our formalization is based on Isabelle/HOL, and we state theorems and definitions following Isabelle’s syntax. For instance,  indicates that  is a function that takes integers and returns elements of type $\alpha$, which is of class  . The type of polynomials over coefficients of type $\alpha$ is denoted by  . In Isabelle, a polynomial $f(x) = \sum _{i=0}^m f_i x^i$ is written as  (the leading coefficient comes last in the list),  denotes the coefficient $f_i$,  the degree *m*, and  the evaluation *f*(*a*) at .

A number *a* is *algebraic* if it is a root of a non-zero integer polynomial *f*. The notion is defined in Isabelle 2018 as follows.

### Definition 1


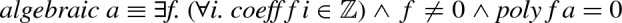


Here the condition that *f* is an integer polynomial is expressed by enforcing the coefficients of *f* to be in the set ${\mathbb {Z}}$, which is of type  . In this definition, the polynomial  and the algebraic number  share the same domain type $\alpha$, which will be instantiated by  or  . Since our motivation is to implement functions that actually operate on algebraic numbers of type  and  , manipulating polynomials in type  leads to a circular dependency. Hence we introduce the predicate  , meaning that a non-zero *integer* polynomial  has  as a root.

### Definition 2


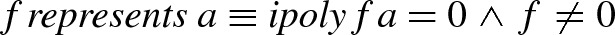


Here,  is an abbreviation for  , and  converts the type of integer polynomials.

We obtain the following alternative characterization of algebraic numbers.

### Lemma 1


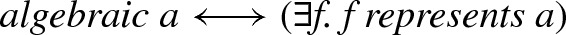


### Unique Representation

An algebraic number can be represented by arbitrarily many polynomials; for instance $\sqrt{2}$ is represented by $f(x) = x^2 - 2$, $g(x) = -x^2 + 2$, $h(x) = x^4 + 2x^3 -4x - 4$, $k(x) = 2x^2 - 4$, etc. However, every algebraic number can be uniquely represented by an integer polynomial which has no non-trivial divisors and a positive leading coefficient. The degree of this unique representative polynomial is called the degree of the algebraic number. For instance, *f* is this unique representative of $\sqrt{2}$, whereas *g* has a negative leading coefficient, and *h* is reducible as $h(x) = f(x) \cdot (x^2 + 2x + 2)$.

Irreducibility of a polynomial often means that it is non-constant and has no non-constant divisor of smaller degree, and in the preliminary version of this work [24] we used such a definition. Isabelle 2018, however, uses the following predicate  for arbitrary commutative rings.

#### Definition 3


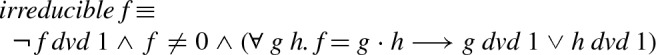


Here  is Isabelle’s notation for divisibility. This definition is stronger than the polynomial-specific version. In particular,  for non-constant integer polynomial *f* demands that *f* is *content-free*, i.e., the GCD of the coefficients of *f* is 1; otherwise, *f* is “reducible” by the GCD. For instance, the integer polynomial *k* above is reducible since $k(x) = (x^2 - 2) \cdot 2$. Note also that the definitions are equivalent on field polynomials.

We adopt this stronger definition in the current work, and formulate the uniqueness statement as follows.

#### Lemma 2


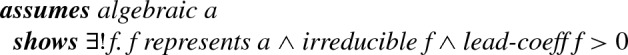


Typical uniqueness results found in the literature (e.g., [11, pp. 700] and [20, pp. 319]) state that there is a unique representative polynomial of the *minimum degree*. Our claim is more useful for computing the unique representative: if we find any irreducible polynomial representing a number, then we do not have to search for other polynomials of lower degree that represent the same number. The typical statement is easily obtained from Lemma 2; actually the irreducible representative polynomial is of the minimum degree.

#### Corollary 1


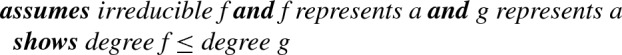


To prove Lemma 2 we first show that polynomials over a *unique factorization domain* (UFD) forms a UFD again. Whereas the class  was (independently) introduced to Isabelle 2016-1 for UFDs, it demands several extra operations, i.e.,  ,  ,  , etc., to derive that polynomials over a UFD form a UFD. We instead define a more general class  : 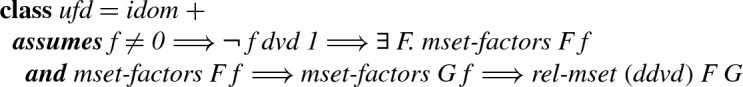 Here the first assumption claims that for every non-zero and non-unit element *f* in the domain, there exists a multiset  of irreducible factors of *f*, denoted by the predicate  . The second assumption claims that for any element *f*, any two irreducible factorizations *F* and *G* of *f* are “associated”, i.e., *F* and *G* contain the same number of factors, $f_1, \dots , f_n$ and $g_1, \dots , g_n$, such that  . Here  is defined as  .

In this general setting we show that polynomials over a UFD form a UFD. 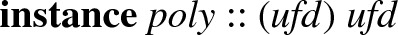 This result is instantly lifted to any multivariate polynomials; if $\alpha$ is of sort  , then so is  , and thus so is  , and so on. This is crucial for formalizing addition and multiplication of algebraic numbers, where we extensively use bivariate polynomials.

We also establish a connection between  and the already existing locale  . Thus we can derive results from  , e.g., that irreducibility and primality are equivalent in UFDs. This yields that for an irreducible integer polynomial *f* with positive leading coefficient, the GCD of *f* and any polynomial *g* is either 1 or *f* itself:

#### Lemma 3


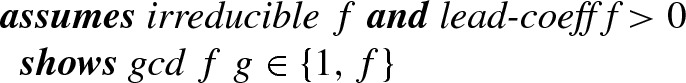


To prove Lemma 2 we further show that the GCD of two integer polynomials stays the same up to a constant factor if we embed ${\mathbb {Z}}$ into ${\mathbb {R}}$ or ${\mathbb {C}}$.

#### Lemma 4


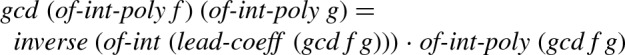


Our proof of Lemma 2 then works as follows: Assume that *f* and *g* are two different, positive and irreducible integer polynomials with a common real or complex root *a*. That is, *f* and *g* as real or complex polynomials have a common factor $x-a$ and hence, their GCD is a non-constant polynomial. On the other hand, the GCD of *f* and *g* as integer polynomials must be 1: it cannot be *f* or *g* itself, since $f \ne g$.

### Unique Representation or Not?

Despite the existence of a unique (and minimal) representative polynomial of an algebraic number, it is *a priori* questionable whether it is a good choice in an implementation to stick to the unique representative polynomials. There is a trade-off between the cost of computing unique representatives from arbitrary representations via polynomial factorization, and the penalty of not using minimal representations in a sequence of operations.

We answer this question experimentally by computing representations of the algebraic numbers $\sum _{i=1}^n \sqrt{i}$ for various *n*. In one configuration we stick to the unique representatives and perform complete polynomial factorization after each addition. In another configuration we only perform the efficient square-free factorization that eliminates duplicate factors.

The result is reported in Table 1, where the computation time *t* and the degree *d* of the representing polynomial is reported as *t* / *d*. Here the benefit of complete factorization is clear; the growth of the degrees is so rapid that manipulating the high-degree polynomials is more costly than applying complete factorization each time.

**Table 1:** Computation time/degree of representing polynomials for $\sum _{i=1}^n \sqrt{i}$

Factorization	$n = 6$	$n = 7$	$n = 8$	$n = 9$	$n = 10$
Square-free	0.054s/64	0.807s/128	19.725s/256	3m19s/384	1h48m/768
Complete	0.019s/8	0.044s/16	0.080s/16	0.080s/16	0.117s/16

Hence we choose irreducible polynomials for representing algebraic numbers; however for those of degree 1, i.e., the rational numbers, there is already an efficient implementation. When implementing a binary arithmetic operation on algebraic numbers, we actually implement two variants: one on a rational and an algebraic number, and another one on two algebraic numbers. The former variant is faster to execute than the more generic latter one. This special treatment for rational numbers explains why there is no measurable difference in Table 1 in the computation time of $\sum _{i=1}^8 \sqrt{i}$ and $\sum _{i=1}^9 \sqrt{i}$: the last addition when computing

$$\begin{aligned} \left( \sum _{i=1}^8 \sqrt{i}\right) + \sqrt{9} = \left( \sum _{i=1}^8 \sqrt{i}\right) + 3 = \ldots \end{aligned}$$

will be the efficient “rational + algebraic”-addition.

## Synthesizing Representative Polynomials

In order to define arithmetic operations over algebraic numbers, the first task is the following: Given polynomials that represent the input numbers, compute a polynomial that represents the output number. In the sequel, we will illustrate the constructions for the various arithmetic operations in ascending difficulty.

### Constants

Obviously, a rational number $a = \frac{n}{d}$ can be represented by $dx - n$.

#### Definition 4



#### Lemma 5



Isabelle’s implementation of the rational numbers ensures that *n* and *d* are coprime and $d\ge 1$. Therefore the polynomial is already positive and irreducible.

#### Lemma 6



### Negation and Inverse

Consider an algebraic number *a* represented as a root of $f(x) = \sum _{i=0}^m f_i x^i$. To represent the unary minus $-a$, the polynomial  , defined as $f(-x)$, i.e., $\sum _{i=0}^m (-1)^i f_i x^i$, does the job.

#### Lemma 7


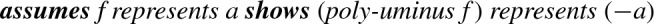


For the inverse $\frac{1}{a}$, it is also not difficult to show that the *reciprocal polynomial*$\sum _{i=0}^m f_i x^{m-i}$, which is defined in Isabelle 2018 as  , has $\frac{1}{a}$ as a root.

#### Lemma 8


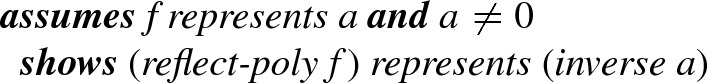


It is beneficial to also show that  and  preserve irreducibility, since otherwise we would have to perform polynomial factorization to maintain the invariant of always working on irreducible polynomials. We argue as follows: Suppose that *f* is irreducible and represents *a*. Clearly  preserves the degree and content; thus if  is reducible, then there is a polynomial *h* of smaller degree that represents $-a$. Since  represents $-(-a) = a$, we obtain a polynomial representing *a* whose degree is smaller than *f*. This contradicts the uniqueness of *f*.

The same argument works also for  , and we formalize the following lemma that generalizes the two.

#### Lemma 9


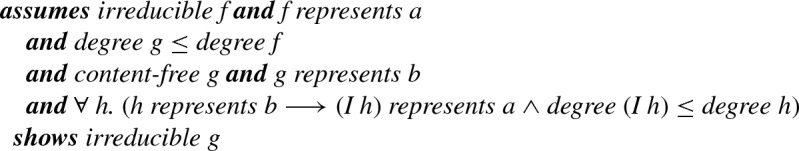


By instantiating *b* in the lemma by $-a$, *g* by  , and *I* by  , we obtain the desired result for  . Similarly we easily obtain the result for  .

#### Lemma 10


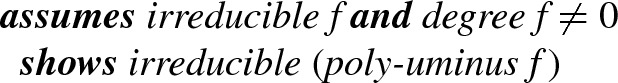


#### Lemma 11


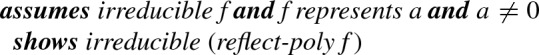


### Multiplication and Addition with Rational Numbers

If we had chosen rational polynomials to represent algebraic numbers, it would be easy to add or multiply a rational number to an algebraic number: when *f* represents *a*, the rational polynomials $f(x - \frac{n}{d})$ and $f(\frac{d}{n} \cdot x)$ represent $a + \frac{n}{d}$ and $a \cdot \frac{n}{d}$, respectively. In our current formalization, however, we work with integer polynomials for efficiency reasons. As neither $f(x - \frac{n}{d})$ nor $f(\frac{d}{n} \cdot x)$ is in general an integer polynomial, we define the polynomials slightly differently.

To represent $a \cdot \frac{n}{d}$, we use the following constant multiple of $f(\frac{d}{n} \cdot x)$:

$$\begin{aligned} n^m \cdot f\left( {\textstyle \frac{d}{n} \cdot x}\right) = \sum _{i=0}^m f_i \cdot d^i \cdot n^{m - i} \cdot x^i \end{aligned}$$

where $f(x) = \sum _{i=0}^m f_i \cdot x^i$. Similarly, for $a + \frac{n}{d}$ we first compute $d^m \cdot f(\frac{1}{d} \cdot x)$, and compose this polynomial and $dx - n$ to obtain $d^m \cdot f(x - {\frac{d}{n}})$. In the following definition, $f \circ _p g$ denotes the polynomial composition *f*(*g*(*x*)), and the monomial $cx^n$ is denoted by  .

#### Definition 5


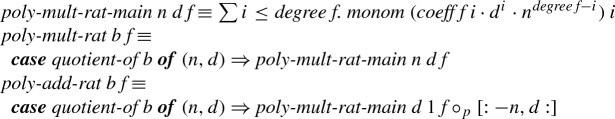


We prove the desired correctness results in a straightforward way.

#### Lemma 12


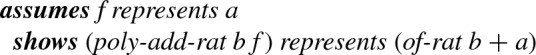


#### Lemma 13


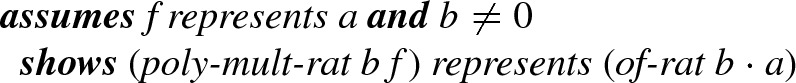


The condition $b \ne 0$ in Lemma 13 stems from the fact that we are essentially performing division. In practice this just demands a special case for $b = 0$, which trivially results in the rational number 0.

Unfortunately both  and  do not preserve irreducibility in terms of Definition 3 in general, since they do not preserve content; e.g., for $f(x) = 2x-3$, the unique representation of $\frac{3}{2}$,  results in the polynomial $2x-6$, which represents 3 but is not content-free. Nevertheless, we only need to eliminate content to obtain irreducibility. We define a function  which divides all coefficients by the content, and additionally ensures a positive leading coefficient. Note that *f* represents *a* if and only if  represents *a*. Since each of the above functions preserves degree, and an inverse operation can be found, we apply Lemma 9 and derive the desired irreducibility results.

#### Lemma 14


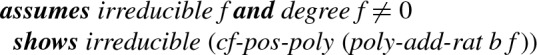


#### Lemma 15


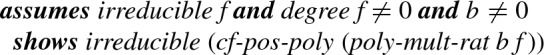


### *n*-th Root

For *n*-th root of *a* represented by $f(x) = \sum _{i=0}^m f_i x^i$, it is easy to see that $f(x^n)$, i.e., $\sum _{i=0}^m f_i x^{ni}$, represents $\root n \of {a}$.

#### Definition 6


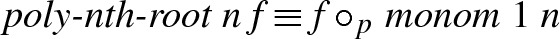


#### Lemma 16


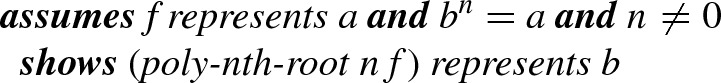


We stated the result for *n*-th roots without using Isabelle’s operations  and  , because they are defined only on types  and  , respectively, but not on a generic field. We easily derive the results for the specific types.

#### Lemma 17


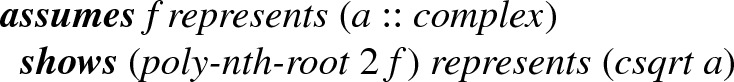


#### Lemma 18


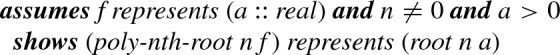


In contrast to previous sections,  does not preserve irreducibility, even though it preserves contents. Consider, e.g.,  applied to $x-64$, the unique representative of 64. The resulting polynomial is $x^4-64$, which can be factored into $(x^2-8)\cdot (x^2 + 8)$. Also for the polynomials obtained from addition and multiplication of two algebraic numbers, we cannot ensure irreducibility in general. We address this issue in Sect. 5.3.

### Addition and Multiplication of Algebraic Numbers

To add or multiply two irrational algebraic numbers *a* and *b*, respectively represented as roots of polynomials *f* and *g*, we must compose non-zero polynomials  and  that have $a + b$ and $a \cdot b$ as a root.

For this purpose the resultant is a well-known solution. The resultant of the polynomials $f(x) = \sum _{i=0}^m f_i x^i$ and $g(x) = \sum _{i=0}^n g_i x^i$ is defined as $\mathrm {Res}_{}(f,g) = \det (S_{f,g})$, where $S_{f,g}$ is the *Sylvester matrix*:Note that if *f* and *g* are univariate, then $\mathrm {Res}_{}(f,g)$ is a constant. We write $\mathrm {Res}_{x}(f(x),g(x))$ for $\mathrm {Res}_{}(f,g)$ when the resolved variable *x* should be clarified. If *f* and *g* are bivariate then $\mathrm {Res}_{y}(f(x,y),g(x,y))$ is a univariate polynomial over *x*.

We first state the desired result for addition. Here,  is defined as the univariate polynomial $\mathrm {Res}_{y}(f(x-y),g(y))$.

#### Lemma 19


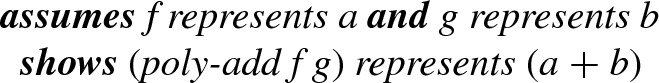


We perform multiplication through division by the inverse, and division as follows:  is defined as $\mathrm {Res}_{y}(f(x\cdot y),g(y))$ and  ensures that *g* does not represent 0, so that in particular $b \ne 0$.

#### Lemma 20


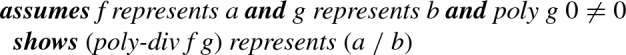


To prove each lemma, we need to prove two claims: the resultant has a desired root, and is a non-zero polynomial. In the next sections we prove each of the claims.

#### Resultant has Desired Roots

For non-constant polynomials *f* and *g* over a commutative ring, we can compute polynomials *p* and *q* such that for arbitrary *x*,

1 $$\begin{aligned} \mathrm {Res}_{}(f,g) = p(x) \cdot f(x) + q(x) \cdot g(x). \end{aligned}$$

To formally prove the result, we first define a function  that operates on the Sylvester matrix. For each *j*-th column except for the last one,  adds the *j*-th column multiplied by $x^{m+n-j}$ to the last column. Each addition preserves determinants, and we obtain the following equation:2Note here that only the last column depends on *x*. We can extract this column using the *Laplace expansion*, which we formalize as follows.

##### Lemma 21


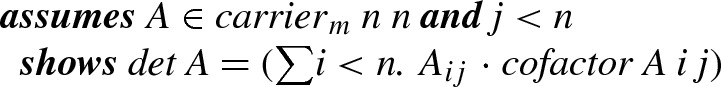


Here  just means that *A* is an $n\times n$ matrix over the considered ring, and *Aij* is defined as , where *B* is the *minor matrix* of *A* obtained by removing the *i*-th row and *j*-th column. Thus we can remove the last column of the matrix *A* in (2), by choosing $j = m+n-1$. Note that then every  is independent from *x*. We obtain *p* and *q* in (1), as $\mathrm {Res}_{}(f,g)$ is represented as follows:

##### Lemma 22


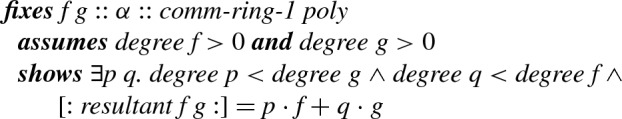


The lemma implies that, if *f* and *g* are polynomials of positive degree with a common root, say $f(a) = g(a) = 0$, then

$$\begin{aligned} \mathrm {Res}_{}(f,g) = p(a) \cdot f(a) + q(a) \cdot g(a) = 0 \end{aligned}$$

The result is lifted to the bivariate case: $f(a,b) = g(a,b) = 0$ implies that $\mathrm {Res}_{y}(f(a,y),g(a,y)) = 0$ for all *a* and *b*.

##### Lemma 23


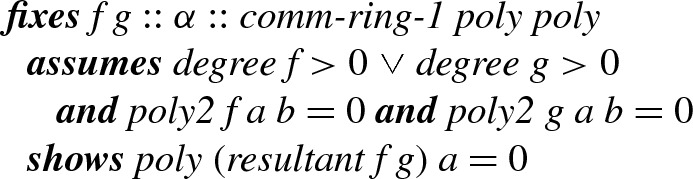


Here,  is our notation for bivariate polynomial evaluation.

Now for univariate non-zero polynomials *f* and *g* with respective roots *a* and *b*, the bivariate polynomials $f(x-y)$ and *g*(*y*) have a common root at $x = a+b$ and $y = b$. Hence, Lemma 23 indicates that the univariate polynomial $\mathrm {Res}_{y}(f(x-y),g(y))$ has $x = a+b$ as a root.

##### Lemma 24


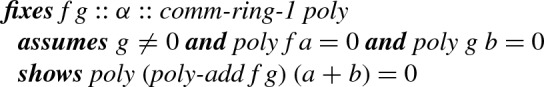


We need a variation of Lemma 24 in which  and  are of type  while  and  are still of type $\alpha$. We prove some homomorphism lemmas to obtain the following:

##### Lemma 25


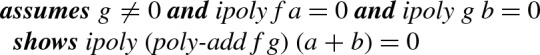


Analogously, if $b \ne 0$, then $f(x\cdot y)$ and *g*(*y*) have a common root at $x = a/b$ and $y = b$.

##### Lemma 26


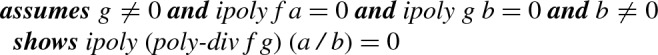


#### Resultant is Non-Zero

Now consider the second claim:  and  are non-zero polynomials. Note that they would otherwise have any number as a root. Somewhat surprisingly, formalizing this claim is more involved than the first one.

We first strengthen Lemma 22, so that *p* and *q* are non-zero polynomials. Here, we require an integral domain  , i.e., there exist no zero divisors.

##### Lemma 27


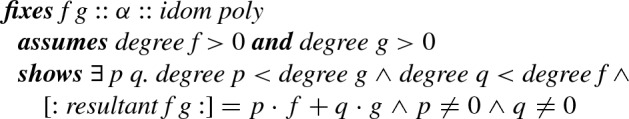


The proof is easy for the case where $\mathrm {Res}_{}(f,g)$ is non-zero: we obtain *p* and *q* using Lemma 22, and it is easy to see that $p \cdot f + q \cdot g$ cannot be a constant if $p = 0$ or $q = 0$, using the constraints on degrees. For the case $\mathrm {Res}_{}(f,g) = 0$, we formalize the classical result that linear equation $A\mathbf {v} = \mathbf {0}$ on an integral domain has a non-zero solution if and only if $\det (A) = 0$. Since resultants are the determinants of Sylvester matrices, from a non-zero solution to $S_{f,g}\mathbf {v} = \mathbf {0}$ one can extract non-zero polynomials *p* and *q* as a solution to $p \cdot f + q \cdot g = 0$.

If $\mathrm {Res}_{}(f,g) = 0$, then from Lemma 27 we have $p \cdot f = - q \cdot g$. In UFDs, this implies that *f* and *g* cannot be *coprime*, i.e., that *f* and *g* have a common factor, since otherwise *f* must divide $-q$, contradicting $\textit{degree}(f) > \textit{degree}(q)$.

The definition of the predicate  in Isabelle 2018 relies on the definition of  . We generalize  as follows in order to state the above for arbitrary UFDs:

##### Definition 7


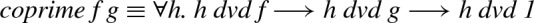


##### Lemma 28


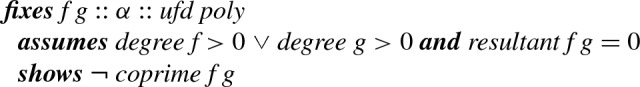


Finally, we reason that $\mathrm {Res}_{y}(f(x-y),g(y))$ and $\mathrm {Res}_{y}(f(x\cdot y),g(y))$ are non-zero polynomials by contradiction. As *f* and *g* are integer polynomials—a UFD— there exist irreducible factorizations: $f = f_1 \cdots f_m$ and $g = g_1 \cdots g_n$. The operation of transforming a univariate polynomial *f*(*x*) to the bivariate $f(x-y)$ is a ring homomorphism, and moreover preserves irreducibility. Thus,

$$\begin{aligned} f(x-y) = f_1(x-y) \cdots f_m(x-y) \end{aligned}$$

is an irreducible factorization. The same property clearly holds for the transformation from *g*(*x*) to *g*(*y*), and we get an irreducible factorization of *g*(*y*):

3 $$\begin{aligned} g(y) = g_1(y) \cdots g_n(y) \end{aligned}$$

Now suppose that $\mathrm {Res}_{y}(f(x-y),g(y)) = 0$. Then Lemma 28 implies that $f(x-y)$ and *g*(*y*) have a common proper factor. Without loss of generality consider an irreducible one *h*(*x*, *y*). In UFDs, this implies that 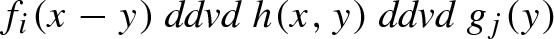 for some $i \le m$ and $j \le n$. By fixing *y*, e.g., to 0, we conclude $f_i(x)$ divides a constant $g_j(0)$, and hence $f_i$ is a constant. This contradicts the assumption that $f_i$ is a proper factor of *f*.

The reasoning is similar for division, but note that the transformation from *f*(*x*) to $f(x \cdot y)$ does not preserve irreducibility: monomial *x* is irreducible but $x \cdot y$ is not. Nevertheless it is a ring homomorphism and we have a (possibly reducible) factorization:

$$\begin{aligned} f(x \cdot y) = f_1(x \cdot y) \cdots f_m(x \cdot y) \end{aligned}$$

Now if $\mathrm {Res}_{y}(f(x\cdot y),g(y)) = 0$, then we obtain an irreducible factor *h*(*x*, *y*) by the same reasoning as above. We still have the irreducible factorization (3) for *g*(*y*), and thus  for some *j*.

As *h*(*x*, *y*) is irreducible and divides $f(x\cdot y)$, it divides some $f_i(x\cdot y)$. Here we need the fact that irreducibility and primality coincide in UFDs. Hence, 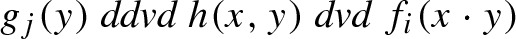 Now we fix *x* to 0. Then we conclude that $g_j(y)$ divides a constant $f_i(0)$, and hence $g_j$ is a constant, leading to a contradiction (the case $f_i(0) = 0$ will ultimately be handled by the assumption that *g* does not represent 0). This proves that  and  are non-zero polynomials, completing our proof of Lemma 19 and Lemma 20.

## Computing the Resultant

Resultants can be computed by first building the Sylvester matrix and then computing its determinant by transformation into row echelon form. A more efficient way to compute resultants has been developed by Brown and Traub: the subresultant polynomial remainder sequence (PRS) algorithm [2, 3].

The algorithm computes $\mathrm {Res}_{}(f,g)$ in the manner of Euclid’s algorithm. It repeatedly performs the polynomial division on the two input polynomials and replaces one input of larger degree by the remainder of the division.

We first consider all computations over the fraction field  , where all division operations are inherently exact. We then prove that intermediate values stay of form $\frac{a}{1}$; that is, we can use a partial division operator  on the integral domain $\alpha$, that satisfies  for $b\ne 0$, but not necessarily  . Therefore, our final implementation works solely on the integral domain, without requiring fraction field operations.

The *j*-*th subresultant* of the polynomials *f* and *g* with $f(x) = \sum _{i=0}^m f_i x^i$ and $g(x) = \sum _{i=0}^n g_i x^i$ is defined as follows:4Note that, in contrast to resultants, the subresultant of polynomials is a polynomial of the same type. However, due to equation (2), $\mathrm {Sub}_{0}(f(x),g(x))$ is actually the constant $\mathrm {Res}_{}(f,g)$.

### Lemma 29



Using the following lemma, we can always assume $\textit{degree}(f) \le \textit{degree}(g)$. In the remainder of this section, we write *m* for $\textit{degree}(f)$ and *n* for $\textit{degree}(g)$.

### Lemma 30



Following Brown and Traub [3], we then formalize the following lemma, showing that a Euclidean algorithm can be used to compute subresultants. As in the Euclidean algorithm, we require a polynomial *h* such that $h = f + b \cdot g$ for some *b*, where $l = \textit{degree}(h) < n$.

### Lemma 31

(Subresultants via Euclidean algorithm)

1. $$j < l \Longrightarrow $$

For non-field polynomials, and in particular bivariate polynomials, polynomial division is not always possible, but *pseudo-division* is: we can find *h* such that $h = d \cdot f + b \cdot g$ for some constant *d*. The following lemma allows us to use pseudo-division instead of division in subresultant computation.

### Lemma 32



Iterated application of pseudo-division results in repeated multiplication with constants $d^{n-j}$, and hence the coefficients of the processed polynomials increase exponentially. One approach to keep the coefficients small is to divide the polynomials by their content in every iteration, as in Collin’s *primitive PRS algorithm* [3, Sect. 4]. We have implemented this approach for the preliminary version [24] of this paper.

This work additionally formalizes the more sophisticated *subresultant PRS algorithm* of Brown and Traub [2, 3]. Here, a constant  —the leading coefficient of a subresultant of the input polynomials—is carried around as an extra argument. It is used to perform exact divisions on the pseudo-remainder polynomials without the necessity to calculate the content in every iteration.

The core of this algorithm is formalized as follows, where  is an Isabelle function that divides a polynomial by a constant.

### Definition 8


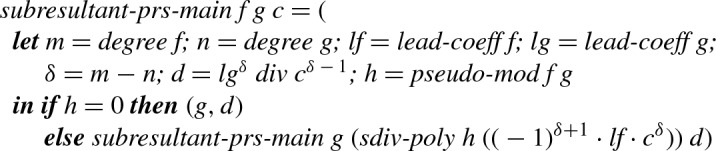


The above function works under the invariant that $n < m$ (so that $\delta - 1 \ge 0$ in Definition 8) and in particular the invariant that all divisions are exact. Thus as the initial step we establish these invariants, and obtain a suitable initial value for  .

### Definition 9


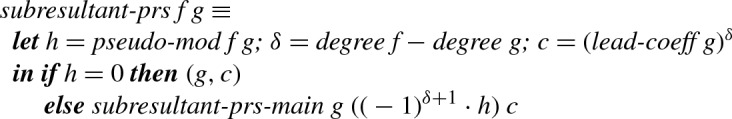


The invocation of  returns a pair (*h*, *d*), where *h* is a scalar multiple of the GCD of  and  , and $\mathrm {Res}_{}(f,g) = d$ if $\textit{degree}(h) = 0$, and $\mathrm {Res}_{}(f,g) = 0$ otherwise.

In addition to the definitions of  and  we develop an optimized implementation in the form of code equations. These optimizations include treating common cases separately, avoiding calculating the same value twice, and replacing expressions like $(-1)^{\delta +1}\cdot h$ by a single negation. We also integrate the efficient calculation of  described by Ducos as *dichotomous Lazard* [8, Sect. 2], but we did not integrate Ducos’ second optimization about the calculation of  in Definition 8.

We define the final function as  , and prove the following correctness result as a code equation:

### Lemma 33


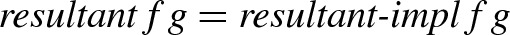


We also define a function  that returns the GCD of two polynomials based on  , and get a correctness result:

### Lemma 34


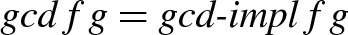


We do not state Lemma 34 as a code equation, since on simple polynomials, e.g., of type  , we experimentally see that the algorithm performs worse than the standard GCD implementation. The algorithm becomes beneficial for multivariate polynomials, e.g.,  .

## Real Algebraic Numbers

In the previous two sections, we have seen how to synthesize a polynomial *f* representing an algebraic number *a* as one of its root. To unambiguously represent *a*, we need to specify which root of *f* is actually *a*. Moreover, we need a concrete representation of real algebraic numbers. Both of these problems are addressed in this section, resulting in a verified implementation of real algebraic numbers.

### Datatypes for Real Algebraic Numbers

For representing real algebraic numbers, we develop a hierarchy of four layers.

**Fig. 2:**
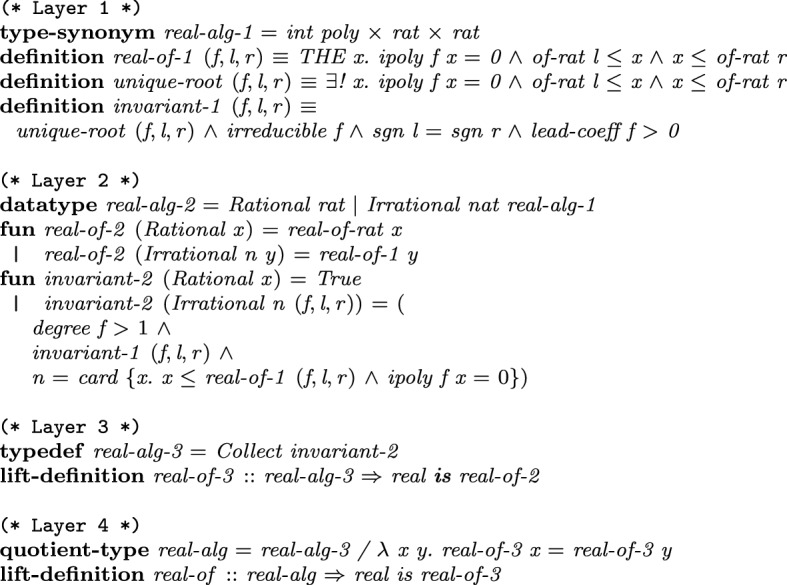
Internal type hierarchy for representing real algebraic numbers, including invariants and conversions to real numbers

**Fig. 3:**
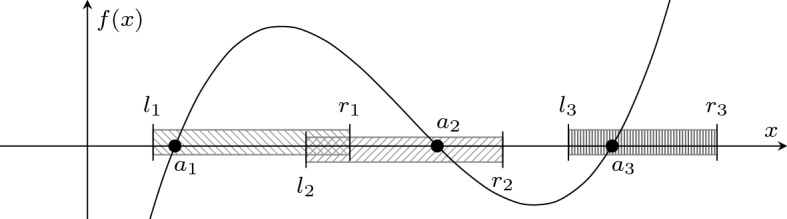
Representing all roots $a_1$, $a_2$, and $a_3$ of some polynomial *f*

On the lowest, Layer  1 (cf. Fig. 2), we define the type  that represents a real algebraic number *a* as a triple (*f*, *l*, *r*), where [*l*, *r*] is a rational interval in which only *a* is a root of *f*. The function  takes such a triple and gives back *a* . This function is defined only on triples that represents exactly one algebraic number, which is ensured as a part of the invariant  . The invariant additionally demands that the representative polynomials are irreducible and have positive leading coefficients, and that the lower- and upper-bounds of the interval have the same sign. For instance, in Fig. 3 the three roots $a_1$, $a_2$, and $a_3$ of *f* are represented by the triples $(f,l_1,r_1)$, $(f,l_2,r_2)$, and $(f,l_3,r_3)$, respectively, as each interval $[l_i,r_i]$ contains exactly one root $a_i$. Note also that the intervals may be overlapping.

Layer 2 introduces the datatype  , which takes a special treatment for rational numbers, so that computations involving only rational numbers will not experience overheads that would arise by manipulating roots of polynomials as in Layer  1. Hence,  demands that the (*f*, *l*, *r*)-form is used only for algebraic numbers of degree at least 2. In  , we additionally store the index of the root, counted from the smallest to the largest.

Layer 3 introduces the type  , which is identical to  but now  is enforced by the type system.

Layer 4 introduces the quotient type  , that identifies different representations of the same number. Hence, the built-in equality of Isabelle/HOL on  corresponds to equality on the represented real numbers. We do not have this property in other layers, since they still permit a number to be represented differently; e.g., $\sqrt{2}$ is encoded by $(x^2 - 2,1,2)$, $(x^2 - 2, 1.4, 1.5)$, etc.

**Fig. 4:**
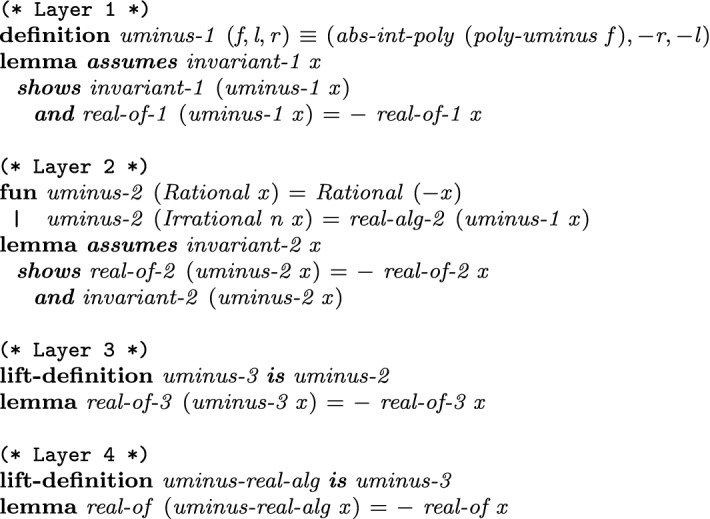
Definitions and properties for negation illustrated for all layers

In each layer we define algebraic operations that are formalized in Sect. 3. Here we take the computation of $-a$ as a first simple example, cf. Fig. 4. Other arithmetic operations like addition require factorization and root separation which will be discussed in Sect. 5.3.

In Layer  1,  computes the new representative polynomial  , and takes $[-r,-l]$ as the new interval. To satisfy the invariant that the leading coefficient is positive,  , which just negates the polynomial if the leading coefficient is negative, is applied. The correctness lemma states that the invariants are preserved and the desired $-a$ is represented.

In Layer  2, we perform a simple case-analysis on whether the represented number *a* is rational or not. If it is, then we use the rational number $-a$, and otherwise invoke  from Layer  1. Afterwards,  is applied. This function converts the triple representation into  by either extracting the rational root if *f* is linear, or by computing the index of the root by invoking Sturm’s method.

Lifting the algorithms and the correctness lemma from Layer  2 to Layer s  3 and 4 is then immediate using Isabelle’s lifting and transfer package [14].

### Comparison and Tightening Intervals

As we work on unique representative polynomials, we can immediately determine the equality of two algebraic numbers in Layer  2: they are equal if the representative polynomials and the root indices match. This test is implemented as follows, and satisfies the corresponding correctness statement. 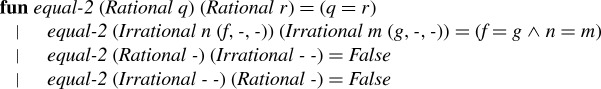


#### Lemma 35


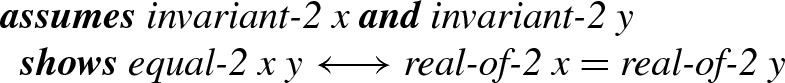


**Fig. 5:**
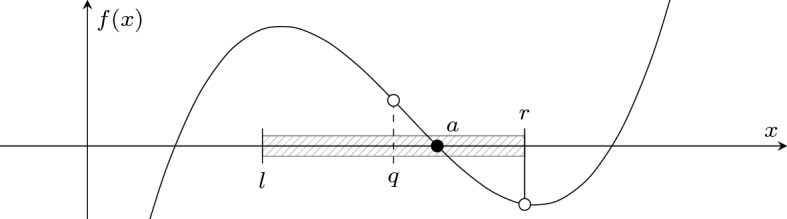
Comparing an algebraic number *a* and a rational number *q*. In this case, we know $q < a$ since *f*(*q*) and *f*(*r*) have different signs

We can efficiently compare an algebraic number *a* and a rational number *q*. Suppose that *a* is irrational and represented by (*f*, *l*, *r*). Then $a = q$ is impossible as *q* is rational, so we want to know whether $a < q$ or $q < a$. It is trivial if $q \notin [l,r]$, and otherwise we compare the signs of *f*(*q*) and *f*(*r*): we know $a < q$ if the signs coincide, and $q < a$ otherwise (see Fig. 5).

#### Definition 10


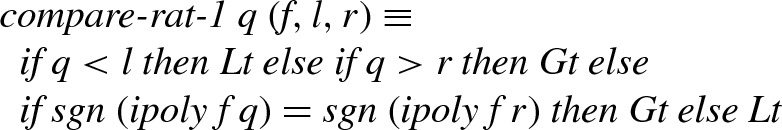


Using this comparison with rational numbers, we can tighten the intervals to arbitrary precision: by taking, e.g., $q = \frac{l+r}{2}$ one can halve the interval to [*l*, *q*] or [*q*, *r*], depending on whether $a < q$ or $q < a$.

Being able to tighten intervals, we can implement the $\left\lfloor a\right\rfloor$ and $\left\lceil a\right\rceil$ operations: tighten the interval of *a* until it contains at most one integer point, and then use the sign-based comparison to determine whether *a* is less or greater than the integer.

**Fig. 6:**
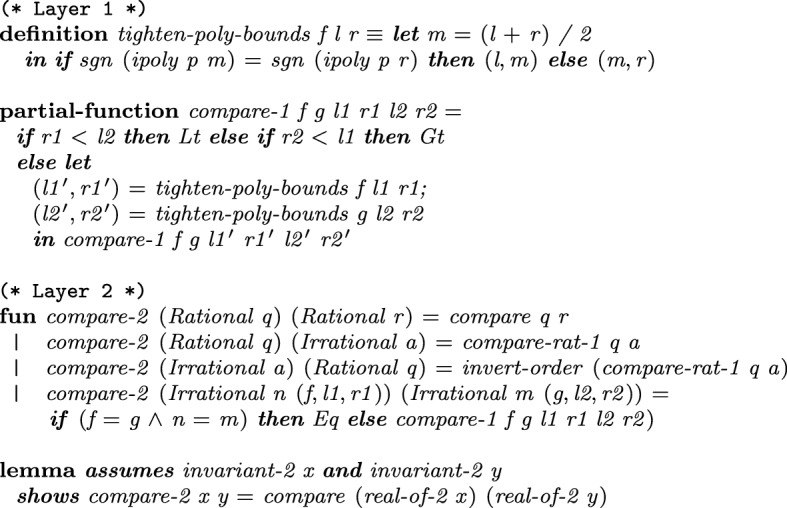
Comparison for the first two layers

We can also compare two irrational algebraic numbers *a* and *b* by tightening intervals. The implementation of the comparison functions^4^ for the first two layers is shown in Fig. 6.

In Layer  1, if *a* and *b* have disjoint intervals, then comparison is trivial. Otherwise  tightens the intervals of *a* and *b* until they become disjoint. The procedure is terminating only if $a \ne b$, since intervals will never become disjoint if $a = b$. Hence Isabelle’s **partial-function** command [16], that allows defining potentially nonterminating procedures, becomes essential. In order to conveniently prove correctness, we define some well-founded relations for inductive proofs, which are reused for various bisection algorithms. For instance, we define a relation based on a decrease in the size of the intervals by at least $\delta$, where $\delta$ is the separation distance, i.e., the minimal distance of two distinct roots of some polynomial.

In Layer  2,  invokes a suitable comparison function depending on rationality: Isabelle’s standard  for two rational numbers,  if exactly one of the inputs is rational, and  for two irrational numbers. Before invoking  ,  first tests equality in order to ensure termination.

### Polynomial Factorization and Root Separation

Recall the invariant of Layer  1: the representing polynomial must be irreducible and have exactly one root in the provided interval. Hence, after synthesizing a polynomial *f* to represent an algebraic number *a*, we must further ensure irreducibility of *f* and provide an interval in which *a* is the only root of *f*.

For unary minus and multiplicative inverse, Lemmas 10 and 11 ensure irreducibility, and moreover the obvious intervals $[-r,-l]$ and $[r^{-1}, l^{-1}]$ work, where [*l*, *r*] is the interval for the input. For other arithmetic operations from Sect. 3, the synthesized polynomial is not generally irreducible, and obviously derived intervals may contain multiple roots.

We first establish irreducibility by a formalized polynomial factorization algorithm [7], and obtain irreducible polynomials $f_1, \cdots , f_n$, such that exactly one of them represents the desired *a*. So the remaining task is to determine which $f_i$ has *a* as a root, and to provide an interval in which *a* is the only root of $f_i$.

We achieve the two goals in one go. Our algorithm maintains: the current interval [*l*, *r*], which contains the desired *a*; a list *F* of candidate polynomials which have at least one root in the interval; and the total number *n* of roots the candidates have in the interval.

The procedure returns if $n = 1$; in this case, *F* contains exactly one polynomial, and this polynomial represents *a*. Otherwise, it tightens [*l*, *r*], and then updates *n* and simultaneously excludes those factors from *F* that have no root in [*l*, *r*]. We will explain later in this section how to count the number of real roots a polynomial *f* has in a given interval.

Note that this procedure terminates only if exactly one candidate polynomial has *a* as a root. Consequently, we again use **partial-function** to define the procedure in Isabelle.

How [*l*, *r*] is tightened depends on the actual operation we are computing. Hence we model the algorithm abstractly using the two functional parameters: 
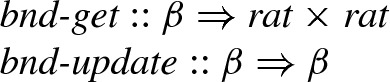
 The type variable $\beta$ represents *states*, which contain sufficient information to maintain intervals. The interval is retrieved by the function  , and  updates one state to another in which a tighter interval is obtained.

For instance, if *a* is the addition of *b* and *c*, represented by $(g,l_b,r_b)$ and $(h,l_c,r_c)$, then the state is a quadruple $(l_b,r_b,l_c,r_c)$,  returns $[l_b + l_c, r_b + r_c]$, and  tightens intervals of *b* and *c* using the bisection algorithm  of Sect. 5.2. Multiplication $a = b \cdot c$ is treated in the same way, except that  returns the interval $[l_b \cdot l_c, r_b \cdot r_c]$; here, the main bisection algorithm for multiplication is only invoked on positive numbers, and a separate algorithm takes care of the signs.

Finally, for computing the *n*-th root $a = \root n \of {b}$ of a positive number *b*, the state is an interval $[l_a,r_a]$ containing *a*, where initially $l_a = \left\lfloor \root n \of {l_b}\right\rfloor$ and $r_a = \left\lceil \root n \of {r_b}\right\rceil$. In every iteration of  , we first compute the rational number $m = \frac{l_a + r_a}{2}$. We then compare *m* with *a* by comparing $m^n$ with *b*. This detour is necessary, since the latter comparison can be computed using  from Sect. 5.2, whereas the former comparison is problematic since *a* is not available. Finally, we update the interval $[l_a,r_a]$ to one of the tighter intervals $[l_a,m]$, [*m*, *m*], or $[m,r_a]$, depending on whether $a < m$, $a = m$, or $m < a$.

We present the correctness statement of the generic factor selection procedure only on Layer  2 where the functions  and  are implicit arguments to  , and where  is iterated function application of  on input  .

#### Lemma 36


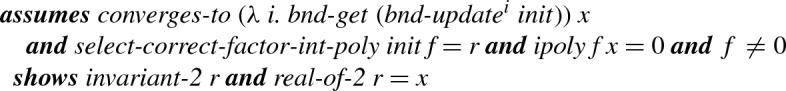


The actual correctness proof in Layer  1 is a rather involved inductive proof; the well-foundedness of the induction relation depends on the convergence of the bounds towards  , and the statement uses 12 invariants that are maintained throughout the proof.

It remains to count how many roots a polynomial *f* has in a given interval. We implement such a root-counting function  using Sturm’s method, with a special treatment for linear polynomials. We extend the existing formalization by Eberl [9], which takes a *real* polynomial and *real* bounds, so that it can be applied on *rational* polynomials with *rational* bounds; nevertheless, the number of *real* roots must be determined. This extension is crucial as we later implement the real numbers by the real algebraic numbers via *data refinement* [13]; at this point we must not yet use real number arithmetic. The correctness of this extension is shown mainly by proving that all algorithms utilized in Sturm’s method can be homomorphically extended. For instance, for Sturm sequences we formalize the following result:

#### Lemma 37


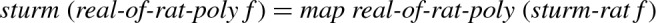


For efficiency, we adapt the algorithm for our specific purpose. Sturm’s method works in two phases: the first phase computes a Sturm sequence, and the second one computes the number of roots by counting the number of sign changes on this sequence for both the upper and the lower bounds of the interval. The first phase depends only on the input polynomial, but not on the interval bounds. Therefore, for each polynomial *f* in the candidate list *F* we precompute the Sturm sequence once, so that when a new interval is queried, only the second phase of Sturm’s method has to be evaluated. This can be seen in the following code equation:

#### Lemma 38


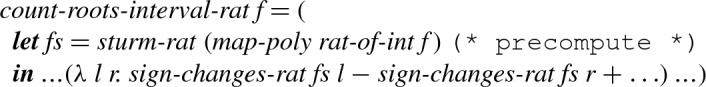


### Implementing Real and Complex Numbers via Real Algebraic Numbers

Having the arithmetic operations on real algebraic numbers, we now provide code equations to implement the real numbers via real algebraic numbers by data refinement, where  is converted into a constructor in the generated code.

#### Lemma 39

(Code lemmas) 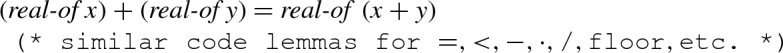


Note that in Lemma 39, the left-hand side of the equality is addition for type  , whereas the right is addition for type  .

As a consequence, Isabelle users now can specify algorithms using algebraic operations on type  and will obtain executable code which uses our verified real algebraic number implementation. Similarly, one can prove a lemma over real numbers like  by evaluation.

To implement complex algebraic numbers, we require nearly nothing: Isabelle implements complex numbers as pairs of real numbers representing the real and imaginary part, and this is possible also in the algebraic setting. Note that a complex number is algebraic if and only if both the real part and the imaginary part are algebraic. Thus, all of the following operations become executable on the complex numbers for free: $+$, −, $\cdot$,  / , $\sqrt{\cdot }$, $=$, and complex conjugate. These operations are already implemented via operations on the real numbers, and computed by real algebraic numbers via data refinement. For instance, complex square roots are computed asAnother approach to implement complex algebraic numbers would be to use *one* representing polynomial in combination with a rectangle in the complex plane to uniquely identify roots, instead of using *two* real algebraic numbers, each requiring its own representing polynomial and interval. As most of our results have been formalized in a generic way, this would become possible if one had replaced the bisection algorithms by similar methods to separate complex roots, e.g., by formalizing results by Kronecker [23, Sect. 1.4.4]. Li provides such a complex root counting algorithm in the AFP [17]. However, his verified implementation currently has some limitations resulting in runtime errors, which makes it at least hard, to use it as a bisection algorithm which must always succeed.

### Displaying Algebraic Numbers

We provide two approaches to display real algebraic numbers, i.e., two functions  of type  . The first one displays an approximative value of an algebraic number *a*. Essentially, the rational number $\frac{\lfloor 1000a \rfloor }{1000}$ is computed and displayed as a string. For instance, $\sqrt{2}$ is displayed as “$\sim$ 1.414”.

The second approach displays an algebraic number without approximation, canonically in form “root #*n* of *f*”. For Layer  4 we have to prove that this approach actually defines a function, i.e., *f* and *n* are uniquely defined by the represented algebraic number. This result easily follows from the invariant that we use irreducible polynomials in combination with their uniqueness, Lemma 2.

Besides this well-definedness result, we also prove *soundness* in the following sense. The function  first invokes a function  where 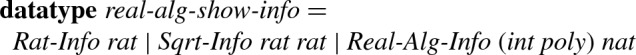 It then converts these intermediate values into strings.

Whereas there is no soundness statement for the final  , we prove the following result for  .

#### Lemma 40


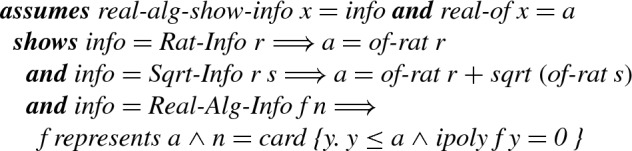


Using  , we define a function for displaying values of type  and then provide its executable implementation via a code equation.

#### Definition 11


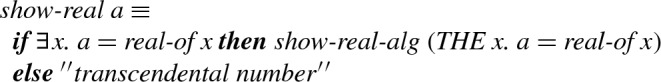


#### Lemma 41


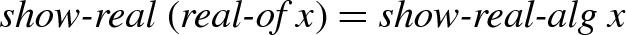


Using  , it is trivial to display complex numbers. Here we only present a simplified definition. The actual definition produces nicer strings if the imaginary part or real part are zero. In the definition, Isabelle’s list-append operator  is used for string concatenation.

#### Definition 12


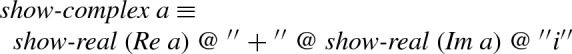


## Real and Complex Roots of Rational Polynomials

In this section, we provide executable functions which identify all real or complex roots of an integer or rational polynomial, as illustrated in Example 1. Without loss of generality we only consider integer polynomials, since every rational polynomial can be converted into an integer polynomial with the same roots, namely by multiplying with the common denominator of the coefficients.

Based on the root finding algorithms, we also provide complete real and complex polynomial factorization algorithms, that work for polynomials with rational coefficients.

### Real Roots of Integer Polynomials

We cannot yet represent the roots of an arbitrary polynomial *f* as  , ...,  as in Example 1, since we have to establish the invariant that *f* is irreducible, and provide an interval for each root.

#### Example 2

The polynomial $f = -14 + 63x + 49x^2 + -490x^3 + 469x^4 + 21x^5 + -126x^6$ has three real roots. The algorithm for computing all roots of *f* will result in the rational number $\frac{1}{3}$ and the first two roots of $g = 2 + 3x + -7x^2 + x^3 + 2x^4$, which are irrational and are the unique roots of *g* in the intervals $[-4,-2]$ and $[-2,0]$.

So, essentially the construction works in three steps:

1. integer polynomial factorization: $f = -7 \cdot (-1 + 3x)^2 \cdot g$ in Example 2
2. construction of initial bounds for roots: all roots of *g* are within $[-8,8]$
3. bisection until one finds intervals for all roots: the roots of *g* are the unique roots of *g* in the intervals $[-4,-2]$ and $[-2,0]$

For the first step we use again the formalized polynomial factorization algorithm. Then we develop an algorithm that, given an irreducible polynomial *f*, produces a list of intervals such that each interval contains exactly one root of *f*, and every root of *f* is contained in one of them. Below we provide more details on the second and third step.

#### Root Bounds

Instead of searching the infinite real space for roots of a polynomial, we start with a closed interval. There are some known bounds on the maximal absolute value of roots of a polynomial. Among them we choose Cauchy’s bound, as it is efficient and easy to formalize, and gives sufficient precision for our purpose.

The *Cauchy boundC*(*f*) of a non-zero polynomial $f(x) = \sum _{i=0}^m f_i x^i$ is defined as follows:

$$\begin{aligned} C(f) = 1 + \frac{\max \{|f_0|,\dots ,|f_{m-1}|\}}{|f_m|} \end{aligned}$$

Then for any root *a* of *f*, we have $|a| \le C(f)$. In the implementation, we do not start with the exact computed bound, which most often is a fraction. Instead, we start with the smallest power of 2 such that $2^n \ge C(f)$. The advantage is that then the bisection algorithm avoids fractions as long as possible. In Example 2, $C(g) = \frac{9}{2}$ and hence, we choose 8 as the initial bound.

##### Definition 13


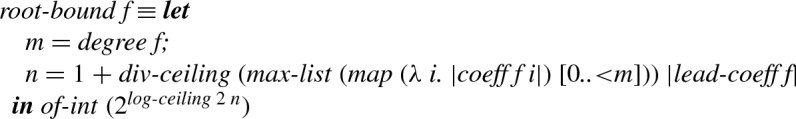


##### Lemma 42


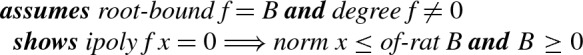


#### Root Separation

Now we separate the roots using a bisection algorithm. The main idea is similar to the interval tightening algorithm in Sect. 5.3. The difference is that here we keep track of all the roots. Hence the algorithm stores two lists: a work list of intervals from which roots of input *f* have to be found, and a result list which stores the already found intervals each containing exactly one root of *f*. Initially the work list is a singleton containing the interval $[-B,B]$ where *B* is the initial bound explained above, and the result list is empty.

In every iteration the algorithm picks up an interval [*l*, *r*] from the work list, and calls the root-counting function  to determine the number *n* of real roots of *f* within this interval. If $n = 0$ then the algorithm throws away this interval and carries on to the next of the work list. If $n = 1$ then a root is identified; the representation (*f*, *l*, *r*) is added to the result list. Finally, if $n > 1$ then the algorithm splits the interval into $[l,\frac{l+r}{2}]$ and $[\frac{l+r}{2},r]$ and pushes them back to the work list. The overlap of the intervals is not problematic, since the bisection algorithm is only invoked on irreducible polynomials of degree at least 2, which cannot have a rational root like $\frac{l+r}{2}$. In particular, the algorithm will return a distinct list of roots.

The bisection algorithm is defined via  for efficiency reasons. The root counting function  , that is obtained after the first phase of Sturm’s method, is passed as a parameter to the main procedure, in order to avoid recomputation. If the algorithm is invoked with an unexpected function, e.g., one that always yields $n = 2$, then it is nonterminating.

We prove that, if correct arguments are passed, then the result of the bisection algorithm is as intended. To this end, we perform well-founded induction on the work list. Here we define $\delta$ as in the bisection algorithm of Sect. 5.2, but now combine the size-measure of intervals with the multiset-extension of a well-founded order [15]. This is required, since if $n > 1$ we replace one interval by two smaller ones.

The final correctness is stated as follows.

##### Lemma 43


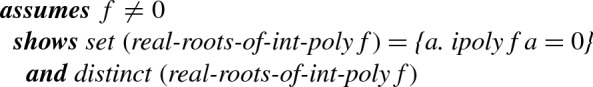


### Complex Roots of Integer Polynomial

In contrast to Sect. 5.4, where complex algebraic numbers are easily implemented via real algebraic numbers, it is not so trivial to develop a complex-number counterpart of  , i.e., a method to identify all complex roots of an integer polynomial *f*.

To identify complex roots, we formalize the following algorithm. It is based on a constructive proof of the fact that a complex number is algebraic if and only if its real and imaginary part are algebraic [22, Corollary 7.3].

- Consider a complex root $a + b{\mathrm {i}}$ of *f* for $a,b \in {\mathbb {R}}$. We have
  $$\begin{aligned} 2 a = (a + b{\mathrm {i}}) + (a - b{\mathrm {i}}) \end{aligned}$$
  and since both $a+b{\mathrm {i}}$ and $a-b{\mathrm {i}}$ are roots of *f*, 2*a* is a root of  . Hence the following polynomial *g* has *a* as a root: 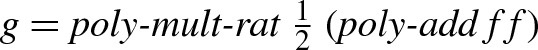 Similarly, $2{\mathrm {i}}\cdot b = (a+b{\mathrm {i}}) - (a-b{\mathrm {i}})$ is a root of  , and as $2{\mathrm {i}}$ is represented by polynomial $4+x^2$, the following polynomial *h* has *b* as a root: 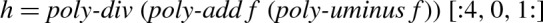
- Let *C* be the set of complex numbers $a+b{\mathrm {i}}$ with  and  . Then *C* contains at least all roots of *f*. Return $\{c \in C.\ f(c) = 0\}$ as the final result.

The actual formalization of  contains several special measures to improve efficiency, e.g., factorizations are performed in between, explicit formulas are used, symmetries are exploited, etc. In particular, we explain how we efficiently compute $\{c \in C.\ f(c) = 0\}$ from *C*.

Since we have now executable complex algebraic operations, one can in principle evaluate *f*(*c*) and test whether it is 0 or not. A drawback of this approach is the demand for manipulating polynomials of high degree. For instance, when testing  in Example 1, complex algebraic numbers like $c^4$ occur. These result in factorization problems for integer polynomials of degree 144.

Instead, we formalize the following algorithm based on interval arithmetic.

- For each $c \in C$, extract the real intervals $I_r$ and $I_i$ from the internal representation of the real and imaginary part, respectively. Use interval arithmetic to test whether $0 \in f( I_r + I_i {\mathrm {i}})$. If 0 is not contained in the interval, remove *c* from *C*.
- If $$|C| = \textit{degree}(f)$$, return *C*.
- Tighten all bounds of *C* so that the extracted intervals will be half of the previous size and start again.

The filter algorithm is formalized on Layer 3, since it is the highest layer which still permits to access the internal interval bounds. Its termination is proven by showing some convergence properties, so that in particular all non-roots are eventually detected and removed. The correctness result of the  looks as in the real case.

#### Lemma 44


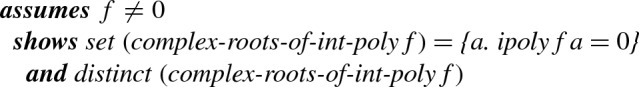


### Factorization of Polynomials over ${\mathbb {C}}$ and ${\mathbb {R}}$

With the help of the complex roots algorithm  and the fundamental theorem of algebra, we further develop two algorithms that factor polynomials with rational coefficients over ${\mathbb {C}}$ and ${\mathbb {R}}$, respectively. Factorization over ${\mathbb {C}}$ is easy: every factor corresponds to a root. Hence, the algorithm and the proof mainly take care of the multiplicities of the roots and factors. Also for factorization over ${\mathbb {R}}$, we first determine the complex roots. Afterwards, we extract all real roots and group each pair of complex conjugate roots. Here, the main work is to prove that for each complex root *c*, its multiplicity is the same as the multiplicity of the complex conjugate of *c*.

## Experiments

Now we experimentally evaluate our implementation. We compare the following three implementations of algebraic numbers:

- *Old version* refers to our verified implementation as described in the preliminary version of this paper [24].
- *New version* refers to our current implementation of algebraic numbers as described in this paper.
- *Mathematica* refers to Wolfram Mathematica 11. Here, we invoke the methods RootReduce and IsolatingInterval in order to obtain the representing polynomial and an interval which uniquely identifies the root, respectively.

Differences with the old version include:

- The type of representing polynomials are now  rather than  .
- We incorporated the verified factorization algorithm [7] while the old version uses an unverified one that does not ensure irreducibility.
- We introduced the sign-based comparison technique (Sect. 5.2) while the old version uses Sturm’s method. Due to this, the old version has to keep Sturm sequences in internal representations while the new version does not.
- We introduced an algorithm that finds the correct factor and a valid interval in one go (Sect 5.3), while the old version performs these tasks sequentially: it first tightens intervals until undesired roots are excluded, and then applies factorization and selects the correct factor.
- We formalized Brown and Traub’s subresultant PRS algorithm (Sect. 4), while the old version uses a variant of Collin’s primitive PRS algorithm.
- We apply interval arithmetic for filtering the complex roots of a polynomial from a list of candidates. In contrast, the old version utilizes algebraic number arithmetic.

All of our implementations have been tested using extracted Haskell code which has been compiled by GHC version 8.2.1 using ghc -O2. The experiments with Mathematica have been conducted within the graphical user interface using Mathematica’s Timing routine. All experiments in this paper have been executed on a 3.2 GHz 8-Core Intel Xeon W with 64 GB of RAM running macOS High Sierra.

**Table 2:** Total time for example computations with algebraic numbers

	Experiment	Old version	New version	Mathematica
(1)	Examples in [18, Fig. 3]	0.032s	0.016s	0.061s
(2)		21.941s	0.207s	0.654s
(3)	$\sum _{i=1}^{10} \sqrt{i}$	0.422s	0.117s	0.070s
(4)	$\sum _{i=1}^6 \root 3 \of {i}$	41.779s	19.902s	0.081s
(5)	$(\sum _{i=1}^{9} \sqrt{i}) - (\sum _{i=1}^{8} \sqrt{i})$	26.459s	2.261s	0.000s

The results of our experiments in Table 2 illustrate that our new implementation is significantly faster than the old implementation.

The big difference in experiment (2) is due to the use of interval arithmetic instead of expensive complex algebraic number computations (Sect. 6.2). For the other experiments, the improvements are mainly due to optimizations of the bisection algorithms and the resultant computation.

In Table 3 we report on detailed profiling information on experiments (4) and (5). The improvements in tightening intervals is due to the sign-based method (Sect. 5.2) and the combined algorithm which tightens intervals and selects correct factors at the same time (Sect. 5.3). In experiment (5) we also see that the subresultant PRS algorithm of Sect. 4 significantly improves the computation time of resultants. As a consequence of our optimizations, polynomial factorization is the main bottleneck of the new implementation.

Table 2 also reveals that there is further room for efficiency improvements in order to compete with the commercial product Mathematica. In particular in Experiment (5), Mathematica can first symbolically simplify the expression to $\sqrt{9}$ which is then easily computed. In contrast, our implementation computes the difference of two real algebraic numbers, each having minimal representatives of degree 16. Still, also our implementation has its benefit: it is formally verified.

**Table 3:** Timing of individual sub-algorithms in percentage of total runtime

Experiment	Algorithm	Old version (%)	New version (%)
(4)	Tightening intervals	59.3	11.5
(4)	Resultant	0.1	0.0
(4)	Factorization	40.1	88.4
(5)	Tightening intervals	13.3	1.5
(5)	Resultant	76.1	35.9
(5)	Factorization	10.7	62.6

## Conclusion

We developed verified algorithms for real and complex algebraic numbers in Isabelle/HOL. These include all the algebraic operations, algorithms to identify complex roots of rational polynomials, and to uniquely present algebraic numbers as strings. The formalization is available to every Isabelle user, and the implementation is available to every programmer as verified Haskell code.

As for future work, a formalization of an equivalent to Sturm’s method for the complex numbers would admit to represent the roots in Example 1 just as root #(1,2,3,4) of  , without the need for high-degree polynomials for the real and imaginary part. Moreover, a more efficient verified polynomial factorization algorithm would be welcome, since this algorithm is currently the most time-consuming part when computing algebraic numbers.

Finally, it would be useful to algorithmically prove that the complex algebraic numbers are algebraically closed, so that one is not restricted to rational coefficients in the factorization algorithms over ${\mathbb {R}}$ and ${\mathbb {C}}$.

## Footnotes

1 See http://math-comp.github.io/math-comp.

2 The suitability test cannot simply evaluate the polynomial on the algebraic point and test whether the result is 0; evaluating at an algebraic point requires the basic arithmetic operations on algebraic numbers, which are the operations we are defining in this work.

3 However, we use a faster computer with 3.2 GHz instead of 2.66 GHz.

4 The formalization differs slightly, since the value of  is carried around for efficiency.

## References

[1] Avanzini, M., Sternagel, C., Thiemann, R.: Certification of complexity proofs using CeTA. In: RTA 2015. pp. 23–39. LIPIcs 36 (2015)

[2] Brown WS (1978). The subresultant PRS algorithm. ACM Trans. Math. Softw..

[3] Brown WS, Traub JF (1971). On Euclid’s algorithm and the theory of subresultants. J. ACM.

[4] Cohen Cyril (2012). Construction of Real Algebraic Numbers in Coq. Interactive Theorem Proving.

[5] Cohen, C., Djalal, B.: Formalization of a Newton series representation of polynomials. In: CPP 2016. pp. 100–109. ACM (2016)

[6] Cohen C, Mahboubi A (2012). Formal proofs in real algebraic geometry: from ordered fields to quantifier elimination. Log. Methods Comput. Sci..

[7] Divasón, J., Joosten, S., Thiemann, R., Yamada, A.: A formalization of the Berlekamp-Zassenhaus factorization algorithm. In: CPP 2017, pp. 17–29 (2017) 10.1007/s10817-019-09526-y PMC7115093 32269396

[8] Ducos L (2000). Optimizations of the subresultant algorithm. J. Pure Appl. Algebra.

[9] Eberl, M.: A decision procedure for univariate real polynomials in Isabelle/HOL. In: CPP 2015. pp. 75–83. ACM (2015)

[10] Eberl, M.: Linear recurrences. Archive of Formal Proofs (Oct 2017), http://isa-afp.org/entries/Linear_Recurrences.html, Formal proof development

[11] von zur Gathen, J., Gerhard, J.: Modern computer algebra, 2nd edn. Cambridge University Press, Cambridge (2003)

[12] Giesl Jürgen, Mesnard Frédéric, Rubio Albert, Thiemann René, Waldmann Johannes (2015). Termination Competition (termCOMP 2015). Automated Deduction - CADE-25.

[13] Haftmann Florian, Krauss Alexander, Kunčar Ondřej, Nipkow Tobias (2013). Data Refinement in Isabelle/HOL. Interactive Theorem Proving.

[14] Huffman Brian, Kunčar Ondřej (2013). Lifting and Transfer: A Modular Design for Quotients in Isabelle/HOL. Certified Programs and Proofs.

[15] Jouannaud JP, Lescanne P (1982). On multiset orderings. Inf. Process. Lett..

[16] Krauss Alexander (2010). Recursive Definitions of Monadic Functions. Electronic Proceedings in Theoretical Computer Science.

[17] Li, W.: Count the number of complex roots. Archive of Formal Proofs (Oct 2017), http://isa-afp.org/entries/Count_Complex_Roots.html, Formal proof development

[18] Li, W., Paulson, L.C.: A modular, efficient formalisation of real algebraic numbers. In: CPP 2016. pp. 66–75. ACM (2016)

[19] Mahboubi Assia (2006). Proving Formally the Implementation of an Efficient gcd Algorithm for Polynomials. Automated Reasoning.

[20] Mishra B (1993). Algorithmic Algebra. Texts and Monographs in Computer Science.

[21] Nipkow, T., Paulson, L., Wenzel, M.: Isabelle/HOL—A Proof Assistant for Higher-Order Logic, LNCS, vol. 2283. Springer (2002)

[22] Niven, I.: Irrational Numbers. No. 11 in Carus Mathematical Monographs, Mathematical Association of America (1956)

[23] Prasolov, V.V.: Polynomials. Springer (2004)

[24] Thiemann René, Yamada Akihisa (2016). Algebraic Numbers in Isabelle/HOL. Interactive Theorem Proving.

[25] Thiemann, R., Yamada, A.: Formalizing Jordan normal forms in Isabelle/HOL. In: CPP 2016. pp. 88–99. ACM (2016)

